# The additive effects of anaemia and transfusion on long-term survival after coronary artery bypass surgery

**DOI:** 10.1093/ejcts/ezad403

**Published:** 2023-12-07

**Authors:** Armando Abreu, José Máximo, Cláudia Almeida, André Lourenço, Adelino Leite-Moreira

**Affiliations:** Department of Surgery and Physiology, Cardiovascular R&D Center—UnIC@RISE, Faculty of Medicine of the University of Porto, Porto, Portugal; Department of Cardiothoracic Surgery, São João University Hospital Center, Porto, Portugal; Department of Surgery and Physiology, Cardiovascular R&D Center—UnIC@RISE, Faculty of Medicine of the University of Porto, Porto, Portugal; Department of Cardiothoracic Surgery, São João University Hospital Center, Porto, Portugal; Department of Anesthesiology, São João University Hospital Center, Porto, Portugal; Department of Surgery and Physiology, Cardiovascular R&D Center—UnIC@RISE, Faculty of Medicine of the University of Porto, Porto, Portugal; Department of Anesthesiology, São João University Hospital Center, Porto, Portugal; Department of Surgery and Physiology, Cardiovascular R&D Center—UnIC@RISE, Faculty of Medicine of the University of Porto, Porto, Portugal; Department of Cardiothoracic Surgery, São João University Hospital Center, Porto, Portugal

**Keywords:** Coronary artery bypass, Anaemia, Blood transfusion, Survival analysis

## Abstract

**OBJECTIVES:**

To compare the independent and combined effects of anaemia and red blood cell transfusion on late survival after isolated coronary artery bypass grafting.

**METHODS:**

Retrospective analysis of 5243 consecutive patients undergoing primary isolated coronary artery bypass grafting, performed from 2000 to 2015, in a Portuguese Academic Hospital. We identified 1649 patients with preoperative anaemia (A^+^) and 1422 patients who received a perioperative transfusion (T^+^)—the 4 possible combinations allowed for the creation of 4 subgroups (A^−^/T^−^, A^−^/T^+^, A^+^/T^−^ and A^+^/T^+^). The primary endpoint was all-cause mortality at 10 years. We employed inverse probability weighting to control for confounding variables.

**RESULTS:**

Thirty-one percent of the patients had preoperative anaemia, and 27.0% had at least one packed red blood cell transfusion. Inverse probability weighting was effective in eliminating differences in all significant baseline characteristics. The primary endpoint of all-cause mortality at 10 years occurred in 568 patients (20.5%) in the A^−^/T^−^ group, as compared with 204 (24.4%) in the A^−^/T^+^ group (hazard ratio, 1.14; 95% confidence interval, 1.00 to 1.31; *P* = 0.053), 358 (33.8%) in the A^+^/T^−^ group (hazard ratio, 1.53; 95% confidence interval, 1.38 to 1.71; *P* < 0.001), 254 (43.6%) in the A^+^/T^+^ group (hazard ratio, 2.25; 95% confidence interval, 1.97 to 2.56; *P* < 0.001).

**CONCLUSIONS:**

This longitudinal, population-level study emphasizes the adverse long-term outcomes of preoperative anaemia and perioperative red blood cell transfusion. It stresses the importance of an evidence-based, multimodal and multidisciplinary approach to conserving blood resources and optimizing outcomes in patients at high risk for transfusion.

**Figure:**
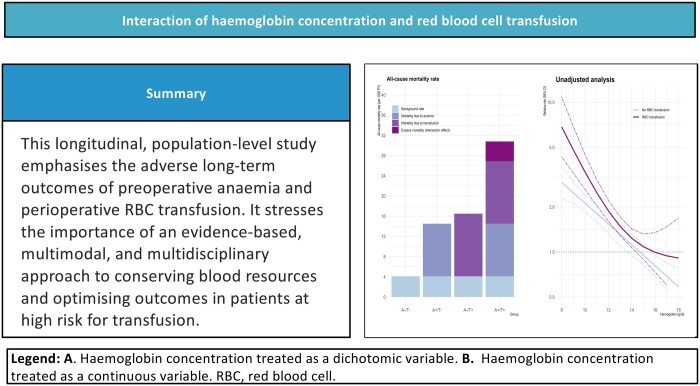

## INTRODUCTION

The World Health Organization defines anaemia as a haemoglobin level of <13 g/dl in men and <12 g/dl in women, translating into a preoperative anaemia prevalence in patients undergoing cardiac surgery from 16% to 54% [1–3]. Hospital-acquired blood loss, iron-deficiency anaemia and anaemia of chronic disease are common causes which are unrelated to the operative procedure itself; nevertheless, the specificities of the surgical practices, such as phlebotomy, intraoperative blood loss and the haemodilution intrinsic to the cardiopulmonary bypass support, might compound the condition [4, 5]. Patients with low haemoglobin concentration have diminished oxygen delivery ability and are prone to end-organ ischaemia. Herein lies the rationale for red blood cell (RBC) transfusion since its primary effect would be increased oxygen delivery and improved organ perfusion.

In the past 20 years, a wealth of data analysed the independent influence of preoperative anaemia and perioperative RBC transfusion on outcomes after cardiac surgery. For instance, 2 meta-analyses [6, 7] established the association between preoperative anaemia and adverse results after surgery. Conversely, several studies described the association of perioperative RBC transfusions with increased long-term mortality [8–11]. However, there is a scarcity of data focusing on the role of the interaction effect of preoperative anaemia and perioperative transfusion on long-term survival.

This article strives to describe the independent and combined effects of preoperative anaemia and perioperative RBC transfusion on long-term survival in a large single-centre inverse probability-weighted (IPW) cohort of patients admitted for isolated coronary artery bypass grafting (CABG). IPW creates a pseudo-population of the treatment and the control groups, with the same covariate distribution as the overall treated and untreated population [12]. This methodology allows us to estimate the average treatment effect in the entire population, the same question asked in a randomized controlled trial.

## MATERIALS AND METHODS

### Ethical statement

The São João University Hospital Centre Ethics Committee approved this research (ID Number 279-13, 25 October 2013) and waived the need for informed consent due to the study’s retrospective nature.

### Study design

This study was a retrospective analysis of an administrative dataset containing all hospitalizations in a level III hospital (University Hospital Centre São João, Porto, Portugal). We included patients undergoing primary isolated CABG from 1 January 2000 to 30 September 2015 (chosen as the cut-off date because of ICD-10-CM implementation).

### Study population

Patients were included in the study if they underwent primary isolated coronary artery bypass surgery [*International Classification of Diseases, Ninth Revision, Clinical Modification* (ICD-9-CM) codes 3610, 3611, 3612, 3613, 3614, 3615, 3616, 3617 or 3619] during the study period. Exclusion criteria included: previous cardiac surgery (ICD-9-CM codes V433 and V4581); concomitant valve replacement or repair (ICD-9-CM codes 3510, 3511, 3512, 3513, 3514, 3520, 3521, 3522, 3523, 3524, 3525, 3526, 3527, 3528 and 3533); concurrent aorta surgery (ICD-9-CM codes 3804, 3814, 3834, 3844, 3845 and 3864); and simultaneous correction of myocardial infarction mechanical complications (ICD-9-CM codes 41410, 4295, 4296, 42971 and 42979).

### Data sources and variables

The corresponding diagnoses and procedures were coded for each hospitalization from the selected patients based on the International Classification of Diseases, 9th Revision, Clinical Modification (ICD-9-CM). Linkage of this dataset to a laboratory data repository allowed for the extraction of each patient's preoperative haemoglobin concentration (in g/dl). Using the World Health Organization cut-off values (men, Hb < 13.0 g/dl; women, Hb < 12.0 g/dl), and whether an RBC transfusion performed, intra- or perioperatively (ICD-9-CM code 9904), 4 treatment groups were thus created (the predictive or independent variable): no anaemia/no RBC transfusion (A^−^/T^−^), no anaemia/RBC transfusion (A^−^/T^+^), anaemia/no RBC transfusion (A^+^/T^−^) and anaemia/RBC transfusion (A^+^/T^+^). Other baseline characteristics were extracted from our Institution’s patient discharge datasets. After obtaining the relevant ICD-9-CM codes, we computed the Charlson Comorbidity Index using the Quan *et al.* coding scheme [13]. We provide definitions of coexisting conditions in Supplementary Material, Table S1.

### Outcomes

The primary outcome variable was long-term all-cause mortality. Through individual Social Security numbers, the discharge dataset allowed linkage to the National Patient Registry (RNU) to ascertain patient life status. Secondary outcomes included perioperative stroke (ICD-9-CM codes 99701 and 99702), prolonged mechanical ventilation (ICD-9-CM codes 9670, 9671 and 9672), acute kidney injury (an increase of over 0.3 mg/dl over baseline creatinine concentration), the length of hospital stay (LOS) and in-hospital mortality.

### Statistical analysis

We present the data as absolute frequencies and percentages for categorical variables and as means and standard deviations for continuous variables. We used the standardized mean difference (SMD) to assess discrepancies in covariates between treatment groups, as it allows for the judgement of the relative balance of variables measured in different units. Values <0.1 indicate a negligible difference in the mean or frequency of a covariate between treatment groups.

We utilized IPW to restrict confounding by indication. IPW makes sense with an active comparator, allowing us to estimate the average treatment effect in the entire population. Making these causal contrasts depends on predicting treatment based on relevant covariates, that is, the propensity score estimation. To this end, we performed covariate balancing propensity score weighting. This method relies on estimating propensity scores using a generalized method of moments and then converting those scores into weights using a formula that depends on the desired estimand. The model included the following variables: sex, age, admission status (scheduled vs unscheduled), disease presentation (stable coronary disease, unstable angina/non-ST-elevation myocardial infarction [NSTEMI] and ST-elevation myocardial infarction [STEMI]), hypertension, diabetes mellitus, hyperlipidaemia, body mass index, smoking history, previous stroke, congestive heart failure, chronic obstructive pulmonary disease, peripheral vascular disease, creatinine clearance (as a surrogate for chronic kidney disease), cancer history and the surgical procedural details (such as whether the procedure was conducted on- or off-pump, the number of internal thoracic arteries used as grafts and the total number of distal anastomosis performed).

We derived weighted logistic regression models with a robust variance estimator with the outcome as the dependent variable and the group on which the propensity score balances (e.g. the treatment group) as the only independent/predictor variable.

Estimates of survival probabilities were calculated using the Kaplan–Meier method and compared with the log-rank test. Follow-up time, described by median and interquartile range (IQR), was obtained using the same estimator by reversing the event indicator so that the outcome of interest became being censored. We employed a weighted Cox proportional hazards regression model with a robust variance estimator to compare long-term mortality between groups.

In order to further clarify the effect of the interaction between anaemia and RBC transfusion, we computed all-cause mortality rates using the number of events and the person-years of follow-up time contributed by the cohort of subjects stratified into subgroups. For transparency, we report the separate effect of each exposure and their joint effect compared to the unexposed group (considered as the background risk).

Finally, we should stress that we did not adjust the 95% confidence intervals (CIs) for multiple comparisons, and inferences drawn from them may not be reproducible.

All the tests of the exposure effect were 2-tailed with an alpha threshold of 0.05. All statistical analyses were performed using R software, version 4.1.3 (R Foundation for Statistical Computing, Vienna, Austria).

## RESULTS

**Figure 1:**
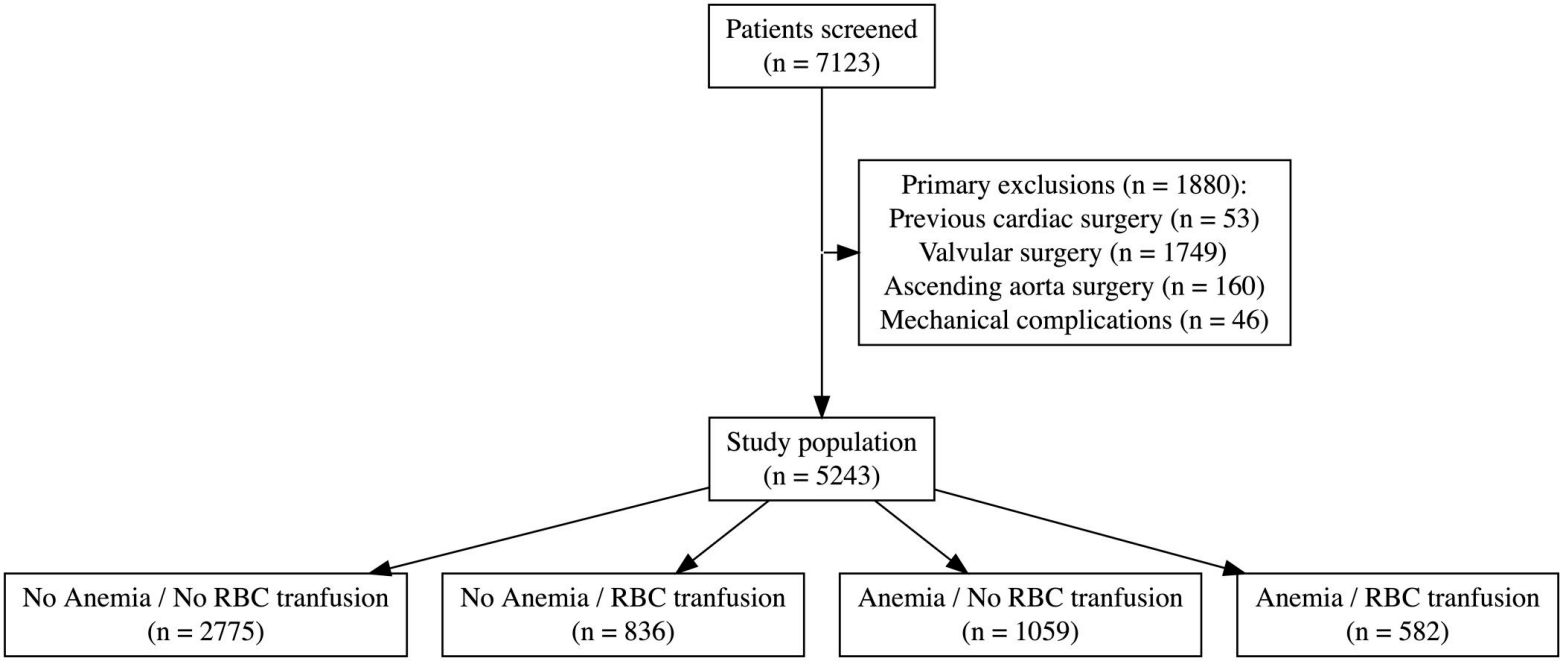
Study population diagram.

Of 7123 patients who underwent CABG during the study period, 5243 were eligible for inclusion and constitute the study population. According to the World Health Organization defined cut-off, 31.3% of the patients had preoperative anaemia, and 27.0% had at least one packed RBC transfusion (Fig. 1). The mean age of the study population was 63.4 (±9.9) years, and 1082 (20.6%) were females.

### Baseline characteristics

Table 1 depicts the baseline characteristics of each one of the study groups. Compared to A^−^/T^−^ patients (mean age 62.31 ± 9.75 years), patients in the A^−^/T^+^ (mean age 63.48 ± 9.71 years, SMD = 0.120), A^+^/T^−^ (mean age 65.52 ± 9.62 years, SMD = 0.332) and A^+^/T^+^ (mean age 67.69 ± 9.61 years, SMD = 0.556) were significantly older. Over 46% of patients in the A^−^/T^−^ group were operated on an unscheduled basis, as compared to 46.5% in the A^−^/T^+^ (SMD = 0.003), 67.5 in the A^+^/T^−^ (SMD = 0.431) and 71.8% (SMD = 0.530) in the A^+^/T^+^ groups.

**Table 1:** Baseline characteristics distributed by study group

Variable	A^−^/T^−^ (*n* = 2775)	A^−^/T^+^ (*n* = 836)	A^+^/T^−^ (*n* = 1059)	A^+^/T^+^ (*n* = 582)
Female sex, *n* (%)	516 (18.6)	190 (22.7)	236 (22.3)	140 (24.1)
Age, mean (SD)	62.31 (9.75)	63.48 (9.71)	65.52 (9.62)	67.69 (9.61)
Admission, *n* (%)				
Scheduled	1480 (53.3)	447 (53.5)	344 (32.5)	164 (28.2)
Unscheduled	1295 (46.7)	389 (46.5)	715 (67.5)	418 (71.8)
Presentation, *n* (%)				
Chronic CAD	1940 (69.9)	516 (61.7)	622 (58.7)	266 (45.7)
UA/NSTEMI	620 (22.3)	241 (28.8)	302 (28.5)	239 (41.1)
STEMI	215 (7.7)	79 (9.4)	135 (12.7)	77 (13.2)
Hypertension, *n* (%)	1761 (63.5)	598 (71.5)	698 (65.9)	478 (82.1)
Diabetes mellitus, *n* (%)				
Non-insulin-treated	865 (31.2)	271 (32.4)	390 (36.8)	241 (41.4)
Insulin-treated	94 (3.4)	50 (6.0)	54 (5.1)	70 (12.0)
Hyperlipidaemia, *n* (%)	1758 (63.4)	568 (67.9)	648 (61.2)	374 (64.3)
BMI, mean (SD)	27.93 (4.01)	27.78 (4.25)	27.70 (4.23)	27.20 (4.33)
Smoking history, *n* (%)				
Never smoked	1663 (59.9)	503 (60.2)	664 (62.7)	346 (59.5)
Former smoker	601 (21.7)	190 (22.7)	227 (21.4)	155 (26.6)
Current smoker	511 (18.4)	143 (17.1)	168 (15.9)	81 (13.9)
Previous stroke, *n* (%)	15 (0.5)	18 (2.2)	8 (0.8)	10 (1.7)
Congestive heart failure, *n* (%)	405 (14.6)	193 (23.1)	216 (20.4)	200 (34.4)
COPD, *n* (%)	177 (6.4)	57 (6.8)	79 (7.5)	61 (10.5)
Creatinine, mean (SD)	1.01 (0.47)	1.02 (0.63)	1.21 (0.91)	1.37 (1.17)
Creatinine clearance, mean (SD)	79.81 (19.17)	78.77 (20.22)	69.71 (23.70)	65.68 (26.02)
Peripheral vascular disease, *n* (%)	148 (5.3)	88 (10.5)	88 (8.3)	107 (18.4)
Previous cancer, *n* (%)	19 (0.7)	5 (0.6)	10 (0.9)	18 (3.1)
CCI, mean (SD)	3.83 (1.48)	4.30 (1.62)	4.51 (1.66)	5.49 (1.81)
Cardiopulmonary bypass, *n* (%)				
OPCAB	1089 (39.2)	288 (34.4)	359 (33.9)	225 (38.7)
ONCAB	1686 (60.8)	548 (65.6)	700 (66.1)	357 (61.3)
Internal thoracic artery use, *n* (%)				
None	79 (2.8)	10 (1.2)	36 (3.4)	18 (3.1)
Single	1891 (68.1)	575 (68.8)	817 (77.1)	424 (72.9)
Bilateral	805 (29.0)	251 (30.0)	206 (19.5)	140 (24.1)
Number of grafts, mean (SD)	2.48 (0.86)	2.55 (0.85)	2.56 (0.86)	2.60 (0.82)

BMI: body mass index; CAD: coronary artery disease; CCI: Charlson Comorbidity Index; COPD: chronic obstructive pulmonary disease; NSTEMI: non-ST-elevation myocardial infarction; ONCAB: on-pump coronary artery bypass; OPCAB: off-pump coronary artery bypass; SD: standard deviation; STEMI: ST-elevation myocardial infarction; UA: unstable angina.

As for the traditional risk factors for atherosclerosis, the prevalence of hypertension was 63.5% in the A^−^/T^−^ group, as compared with 71.5% (SMD = 0.173) in the A^−^/T^+^, 65.9% (SMD = 0051) in the A^+^/T^−^ and 82.1% (SMD = 0.429) in the A^+^/T^+^ groups. Likewise, the prevalence of diabetes mellitus was 34.6% in the A^−^/T^−^, 38.4% (SMD = 0.131) in the A^−^/T^+^, 41.9% (SMD = 0.159) in the A^+^/T^−^ and 53.4% (SMD = 0.446) in the A^+^/T^+^ groups.

Concerning associated comorbidities, congestive heart failure was present in 14.6% of A^−^/T^−^ patients, 23.1% (SMD = 0.218) of A^−^/T^+^, 20.4% (SMD = 0.153) of A^+^/T^−^ and 34.4% in the A^+^/T^+^ groups. Similarly, the mean creatinine clearance, a surrogate for chronic kidney disease, was 79.81 ± 19.17 ml/min in A^−^/T^−^ patients, as compared to 78.77 ± 20.22 in A^−^/T^+^ (SMD = 0.053), 69.71 ± 23.70 in A^+^/T^−^ (SMD = 0.468) and 65.68 ± 26.02 (SMD = 0.618) in the A^+^/T^+^ groups. Peripheral vascular disease was present in 5.3% of A^−^/T^−^ patients, 10.5% (SMD = 0.193) of A^−^/T^+^, 8.3% (SMD = 0.118) of A^+^/T^−^ and 18.4% (SMD = 0.412) of A^+^/T^+^ patients. Correspondingly, the Charlson Comorbidity Index was 3.83 ± 1.48 in A^−^/T^−^ patients, as opposed to 4.30 ± 1.62 (SMD = 0.305) in A^−^/T^+^, 4.51 ± 1.66 (SMD = 0.435) in A^+^/T^−^ and 5.49 ± 1.81 (SMD = 1.01) in the A^+^/T^+^ patients.

Regarding the technical details of the surgical procedure, despite the slight differences recorded, they did not appear to follow the above-noted gradient.

**Figure 2:**
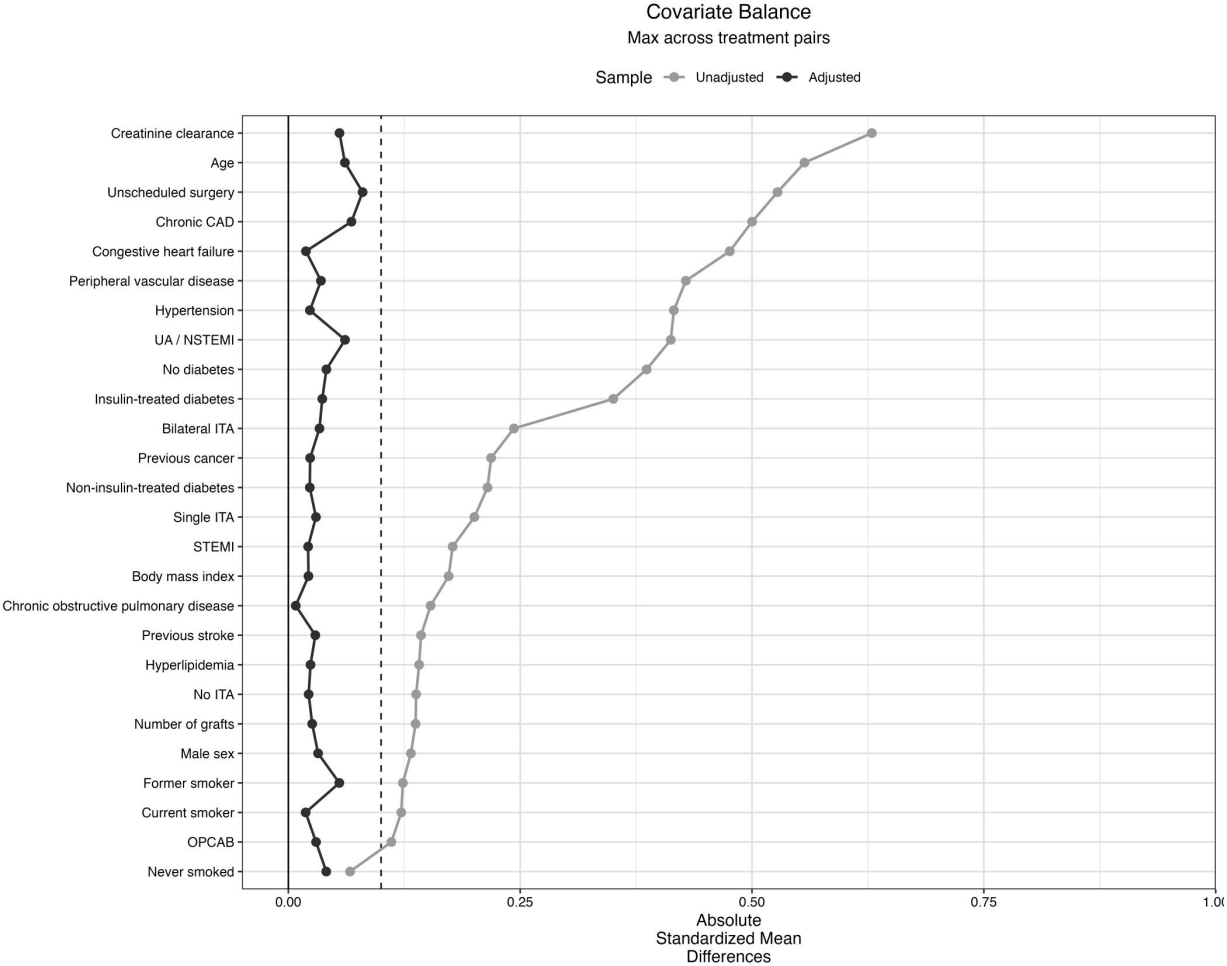
Love plot illustrating covariate balance assessment.

IPW effectively eliminated differences in all baseline characteristics (Fig. 2), as revealed by SMD values below 0.10.

### In-hospital outcomes

When examining predefined secondary outcomes (Table 2), the odds ratio (OR) for perioperative stroke was 3.85 (95% CI, 1.87–8.03; *P* < 0.001) in the A^−^/T^+^ group, 1.69 (95% CI, 0.70–3.85, *P* = 0.223) in the A^+^/T^−^ group and 3.10 (95% CI, 1.28–7.09, *P* = 0.009) in the A^+^/T^+^ group, as compared to the reference group, A^−^/T^−^, in the unadjusted analysis. However, in the adjusted analysis, these differences fell away: the OR was 2.13 (95% CI, 0.97–4.67; *P* = 0.059) in the A^−^/T^+^ group, 1.37 (95% CI, 0.57–3.27, *P* = 0.477) in the A^+^/T^−^ group and 1.46 (95% CI, 0.53–4.02, *P* = 0.464) in the A^+^/T^+^ group, as compared to the reference group, A^−^/T^−^. These results suggest that factors other than preoperative anaemia or RBC transfusion could explain perioperative stroke more reasonably.

**Table 2:** In-hospital outcomes: unadjusted and weighted

Outcome	A^−^/T^−^ (*n* = 2775)	A^−^/T^+^ (*n* = 836)	A^+^/T^−^ (*n* = 1059)	A^+^/T^+^ (*n* = 582)
Stroke, OR (95% CI)				
Unadjusted	Reference	3.85 (1.87, 8.03)	1.69 (0.70, 3.85)	3.10 (1.28, 7.09)
Adjusted	Reference	2.13 (0.97, 4.67)	1.37 (0.57, 3.27)	1.46 (0.53, 4.03)
Prolonged ventilation, OR (95% CI)				
Unadjusted	Reference	3.13 (2.19, 4.47)	1.64 (1.10, 2.41)	3.49 (2.37, 5.11)
Adjusted	Reference	2.85 (1.94, 4.20)	1.36 (0.89, 2.11)	2.81 (1.74, 4.53)
AKI, OR (95% CI)				
Unadjusted	Reference	1.10 (0.90, 1.36)	1.78 (1.50, 2.12)	2.09 (1.70, 2.58)
Adjusted	Reference	1.09 (0.87, 1.37)	1.48 (1.23, 1.78)	1.38 (1.07, 1.78)
LOS (days), Δ estimate (95% CI)				
Unadjusted	Reference	1.45 (0.78, 2.12)	1.98 (1.36, 2.12)	4.07 (3.29, 4.84)
Adjusted	Reference	1.20 (0.08, 2.32)	0.70 (0.14, 1.27)	1.24 (0.41, 2.07)
Mortality, OR (95% CI)				
Unadjusted	Reference	2.40 (1.21, 4.64)	2.52 (1.35, 4.69)	6.13 (3.43, 11.1)
Adjusted	Reference	1.51 (0.72, 3.16)	1.12 (0.56, 2.24)	2.03 (1.03, 4.02)

AKI: acute kidney injury; CI: confidence interval; LOS: length of hospital stay; OR: odds ratio.

Regarding prolonged postoperative mechanical ventilation, the OR was 3.13 (95% CI, 2.19–4.47; *P* < 0.001) in the A^−^/T^+^ group, 1.64 (95% CI, 1.10–2.41, *P* = 0.013) in the A^+^/T^−^ group and 3.49 (95% CI, 2.37–5.11, *P* < 0.001) in the A^+^/T^+^ group, as compared to the reference group, A^−^/T^−^, in the unadjusted analysis. In the adjusted analysis, the OR was 2.85 (95% CI, 1.94–4.20; *P* < 0.001) in the A^−^/T^+^ group, 1.37 (95% CI, 0.89–2.11, *P* = 0.156) in the A^+^/T^−^ group and 2.81 (95% CI, 1.74–4.53, *P* < 0.001) in the A^+^/T^+^ group, as compared to the reference group, A^−^/T^−^. These results suggest that RBC transfusion, but not anaemia itself, might be responsible for the protracted mechanical ventilation times.

Concerning acute kidney injury, the OR was 1.10 (95% CI, 0.90–1.36; *P* = 0.349) in the A^−^/T^+^ group, 1.78 (95% CI, 1.50–2.12, *P* < 0.001) in the A^+^/T^−^ group and 2.10 (95% CI, 1.70–2.58, *P* < 0.001) in the A^+^/T^+^ group, as compared to the reference group, A^−^/T^−^, in the unadjusted analysis. In the adjusted analysis, the OR was 1.09 (95% CI, 0.87–1.37; *P* = 0.431) in the A^−^/T^+^ group, 1.48 (95% CI, 1.23–1.78, *P* < 0.001) in the A^+^/T^−^ group and 1.38 (95% CI, 1.07–1.78, *P* = 0.013) in the A^+^/T^+^ group, as compared to the reference group, A^−^/T^−^. Again, these results suggest that anaemia might be responsible for the acute kidney injury, not RBC transfusion.

In regard the length of hospital stay, it was 1.45 (95% CI, 0.78–2.12; *P* < 0.001) days longer in the A^−^/T^+^ group, 1.98 (95% CI, 1.36–2.59, *P* < 0.001) days longer in the A^+^/T^−^ group and 4.07 (95% CI, 3.29–4.84, *P* < 0.001) days longer in the A^+^/T^+^ group, as compared to the reference group, A^−^/T^−^, in the unadjusted analysis. On the other hand, after extensive covariate adjustment, the length of hospital stay was 1.20 (95% CI, 0.08–2.32; *P* = 0.035) days longer in the A^−^/T^+^ group, 0.70 (95% CI, 0.14–1.27, *P* = 0.015) days longer in the A^+^/T^−^ group and 1.24 (95% CI, 0.41–2.07, *P* = 0.003) days longer in the A^+^/T^+^ group, as compared to the reference group, A^−^/T^−^.

About in-hospital mortality, the OR was 2.40 (95% CI, 1.21–4.64; *P* = 0.010) in the A^−^/T^+^ group, 2.52 (95% CI, 1.35–4.69, *P* = 0.003) in the A^+^/T^−^ group and 6.13 (95% CI, 3.43–11.1, *P* < 0.001) in the A^+^/T^+^ group, as compared to the reference group, A^−^/T^−^, in the unadjusted analysis. However, in the adjusted analysis, the OR was 1.51 (95% CI, 0.72–3.16; *P* = 0.274) in the A^−^/T^+^ group, 1.12 (95% CI, 0.56–2.24, *P* = 0.739) in the A^+^/T^−^ group and 2.03 (95% CI, 1.03–4.02, *P* = 0.042) in the A^+^/T^+^ group, as compared to the reference group, A^−^/T^−^.

### Long-term survival

**Figure 3:**
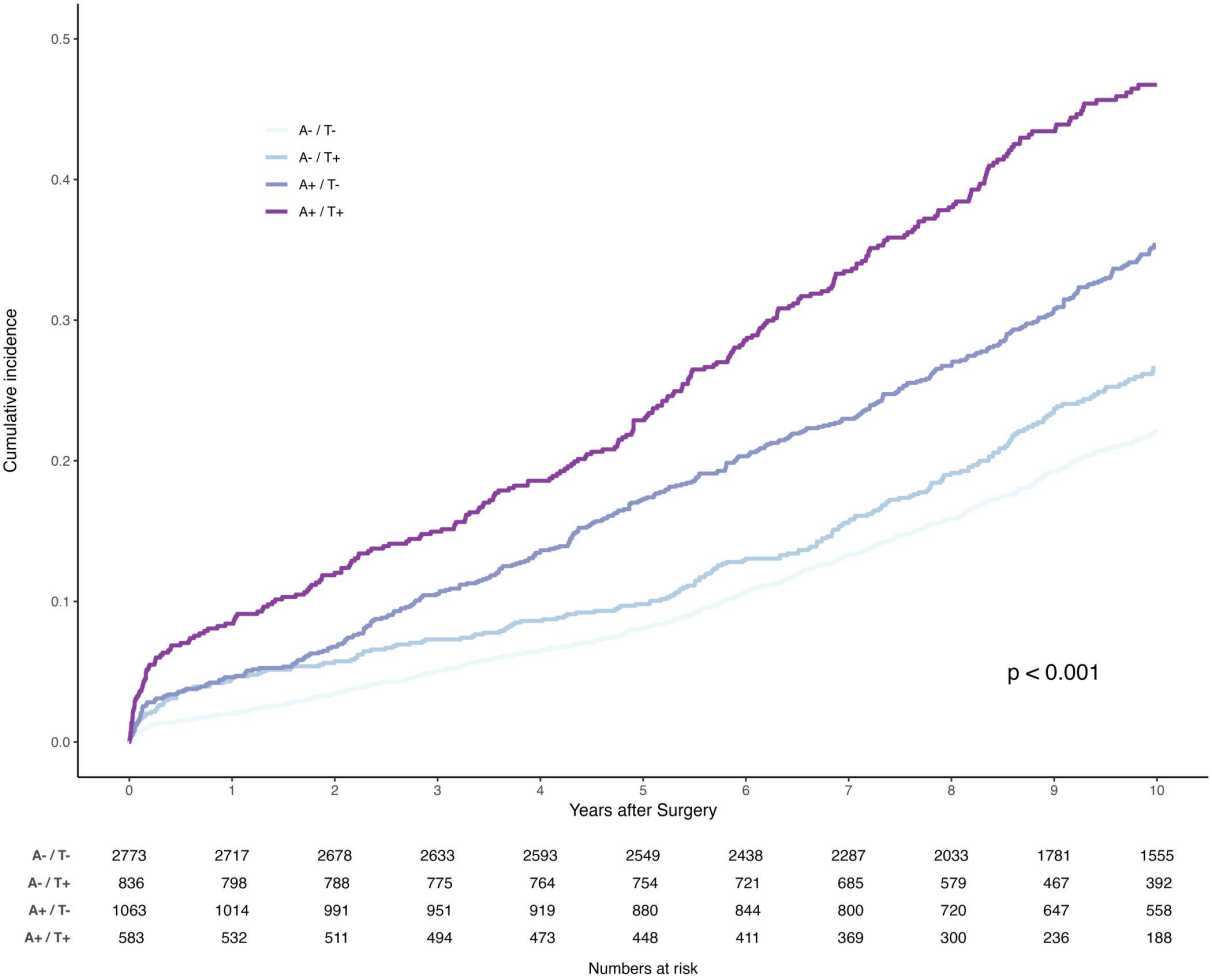
Long-term survival in unweighted cohort (Kaplan–Meier method).

**Figure 4:**
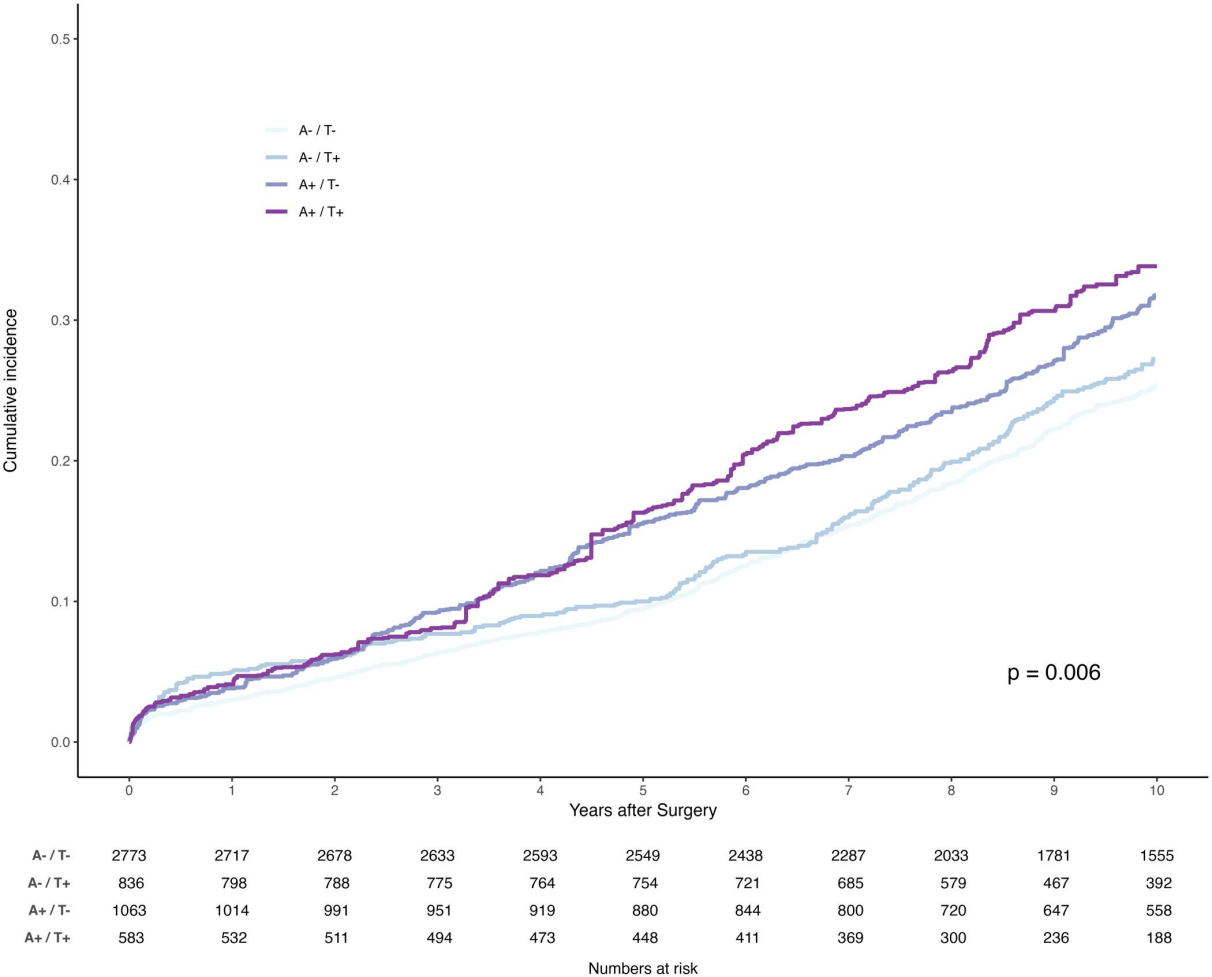
Long-term survival in inverse probability weighted cohort.

Follow-up was 99.71% complete (15 patients with unidentified vital status). The median follow-up time was 12.80 (IQR, 9.52–16.62) years: 12.90 (IQR, 9.60–17.18) years for the A^−^/T^−^ group, 11.83 (IQR, 8.82–14.57) years for the A^−^/T^+^ group, 13.81 (IQR, 10.90–18.67) years for the A^+^/T^−^ group and 11.16 (IQR, 8.75–13.95) years for the A^+^/T^+^ group. The primary endpoint of all-cause mortality at 10 years occurred in 568 patients (20.5%) in the A^−^/T^−^ group, as compared with 204 (24.4%) in the A^−^/T^+^ group (hazard ratio, 1.14; 95% CI, 1.00 to 1.31; *P* = 0.053), 358 (33.8%) in the A^+^/T^−^ group (hazard ratio, 1.53; 95% CI, 1.38 to 1.71; *P* < 0.001), 254 (43.6%) in the A^+^/T^+^ group (hazard ratio, 2.25; 95% CI, 1.97 to 2.56; *P* < 0.001). Thirty-day, 1-, 5- and 10-year survival rates were 99.3%, 98.0%, 91.9% and 77.9% in the A^−^/T^−^ group, 98.6%, 95.4%, 90.2% and 73.2% in the A^−^/T^+^ group, 98.6%, 95.4%, 82.9% and 64.8% in the A^+^/T^−^ group and 96.7%, 91.6%, 77.4% and 53.4% in the A^+^/T^+^ group. Figure 3 depicts the unweighted survival function plot for all groups. Figure 4 illustrates the weighted survival function plot for all groups. Both reveal the difference in long-term survival between groups.

### Interaction of haemoglobin and RBC transfusion

**Figure 5:**
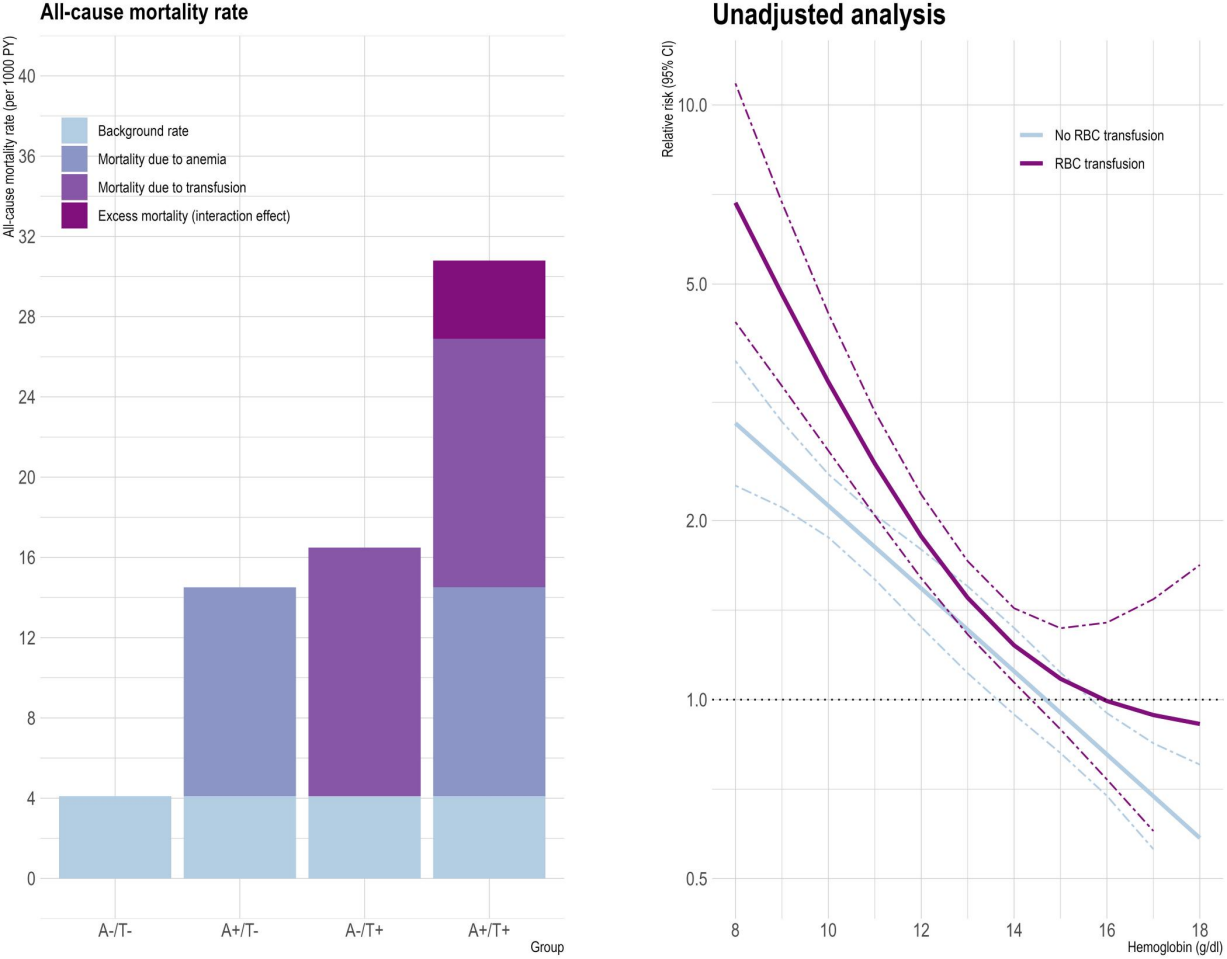
Interaction of haemoglobin concentration and red blood cell transfusion. Haemoglobin concentration treated as a dichotomic variable (left panel). Haemoglobin concentration treated as a continuous variable (right panel).

The all-cause mortality rate was lowest in subjects without anaemia and RBC transfusion (the background rate of 4.10/1000 person-years) (Fig. 5A). The mortality rate in subjects with anaemia (the background rate plus the additional effect of anaemia of 14.5/1000 person-years) was substantially increased. Likewise, the mortality rate in subjects with RBC transfusion (the background rate plus the additional effect of RBC transfusion of 16.5/1000 person-years) was further increased. On top of the combined effect of background, anaemia and RBC transfusion, an additional effect was present. This excess effect of 3.9/1000 person-years is termed the interaction effect.

This interaction effect was again noticeable when the haemoglobin concentration was modelled as a continuous variable, using a natural spline with 2 knots to express its complete flexibility (interaction term, *P* = 0.005) (Fig. 5B).

## DISCUSSION

This longitudinal, population-level evaluation of the independent and combined effects of preoperative anaemia and perioperative RBC transfusion on long-term survival should highlight several key points. First, the basis for perioperative stroke should lie in explanations other than anaemia or RBC transfusion. Prolonged mechanical ventilation appears associated with RBC transfusion but not anaemia; conversely, acute kidney injury seems to result from anaemia, not RBC transfusion. Both could justify the extended length of hospital stay described. On the other hand, only the combined effects of anaemia and RBC transfusion seem to affect short-term mortality. Second, with a median follow-up time exceeding 10 years, preoperative anaemia and perioperative RBC transfusion independently impact late mortality. Finally, it was possible to quantify the interaction effect of the combined anaemia and RBC transfusion on long-term survival.

Regarding immediate postoperative outcomes, a possible explanation for our results might be that preoperative anaemia might be associated with inadequate oxygen delivery ability, impaired tissue oxygenation and subsequent organ dysfunction. This mechanism could justify the increased incidence of postoperative acute kidney injury. On the other hand, RBC transfusion-induced transfusion-related acute lung injury should offer a plausible explanation for increased mechanical ventilation duration. Contrarily, the low event rate of perioperative stroke [*n* = 48 (0.91%)] could undermine our ability to detect significant differences between predefined groups. These, and perhaps other specific complications of cardiac surgery, should explain the extended length of hospital stay described in this study cohort. Finally, only the combined effects of preoperative anaemia and RBC transfusion are associated with an increased risk of in-hospital mortality. Again, with an in-hospital mortality rate of 1.56% (*n* = 82), sample size issues might offer a credible justification for detecting smaller differences between study groups.

The long-term survival impact of anaemia and RBC transfusion appeared to be additive. The effect of preoperative anaemia, compounded by phlebotomy, perioperative blood loss or the haemodilution associated with cardiopulmonary bypass, should extend itself well beyond hospital discharge [14]. Its negative influence exerted over prolonged periods has a well-known association with reduced long-term survival. However, the precise pathophysiological mechanism is not clear. One possible explanation could be the increased haemodynamic overload on the left ventricle, resulting in its remodelling and failure, with an increased mortality risk. Additionally, anaemia is a significant risk factor in ischaemic heart disease, and it correlates with advanced ischaemic heart disease, chronic heart failure, rhythm disturbance, and higher mortality rate [15]. Furthermore, anaemia is associated with higher blood pressure values, and lower dipping status in hypertensive patients [16], thus contributing to an increased cardiovascular risk. Finally, a low haematocrit means hypoxia and cerebral ischaemia; this boosted blood flow and turbulence might enable the migration of a thrombus and embolism [17].

On the other hand, RBC transfusion has profound immunomodulatory effects that render the recipients vulnerable to infection, coronary graft failure, progression of atherosclerosis, malignancy and reactivation of latent viruses [18–20]. Surprisingly, these consequences persist long after the index transfusion, affecting late mortality [21].

As anaemia frequently triggers RBC transfusion, both circumstances may strike the same patient synchronously. Through the distinct mechanisms mentioned above, they take their toll on late survival [11, 22]. Besides, there seems to be a cooperative effect resulting from their interaction. Demonstrating and quantifying this additional risk lends further insight into the research question.

Several observational studies demonstrated the adverse outcomes of preoperative anaemia [2, 3, 23–30]. For instance, Karkouti *et al.* [31] reported the results from a multicentre cohort study that included 3500 patients (67% of patients underwent isolated CABG). Although their definition of preoperative anaemia (haemoglobin < 12.5 g/dl) differed from ours, as well as the methodology employed to control for confounders (multivariable logistic regression and propensity-score matching), the adjusted OR for the composite end-point of in-hospital death, stroke or acute kidney injury was 1.8 (95% CI, 1.2 to 2.7). Similarly, Padmanabhan *et al.* [7] documented the association of preoperative anaemia with adverse outcomes after cardiac surgery in their meta-analysis of over 114 000 patients. While the different studies utilized different anaemia definitions and study populations, their combined analysis revealed increased mortality (OR, 2.74; 95% CI, 2.32–3.24; *P* < 0.001), acute kidney injury (OR, 3.13; 95% CI, 2.37–4.12; *P* < 0.001), stroke (OR, 1.46; 95% CI, 1.24–1.72; *P* < 0.001) and infection (OR, 2.65; 95% CI, 1.98–3.55; *P* < 0.001). They found no statistically significant association between mortality and RBC transfusion (OR, 1.35; 95% CI, 0.92–1.98; *P* = 0.12), albeit the severe heterogeneity in the estimate (*I*^2^ = 83.7%). Interestingly, the authors support the addition of preoperative anaemia to future risk prediction models and as a target for risk modification.

Padmanabhan *et al.* [32], in a single-centre retrospective analysis of 1170 propensity-matched pairs of patients, showed that preoperative anaemia was independently associated with long-term mortality, regardless of whether or not patients received RBC transfusion. They found no association between RBC transfusion and mortality. Furthermore, there was no interaction between preoperative anaemia and blood transfusion on long-term mortality. Differently, in an observational cohort study conducted at a university hospital in Germany that included 3131 patients, von Heymann *et al.* [30] employed multivariate Cox regression analyses to adjust for confounding. Their results indicate that both the severity of preoperative anaemia (mild anaemia: hazard ratio, 1.441; 95% CI, 1.201–1.728; severe anaemia: hazard ratio, 1.805; 95% CI, 1.336–2.440) and intraoperative transfusion (hazard ratio, 1.340; 95% CI, 1.109–1.620) were associated with decreased long-term survival. The authors conclude that long-term survival was worse in anaemic patients receiving intraoperative RBC transfusion. Finally, Schwann *et al.* [21] reported on over 250 000 patients above 65 years undergoing isolated CABG. Using multivariate Cox regression to control for confounding variables, the authors note that both preoperative and intraoperative anaemia are associated with increased mortality, albeit only marginally. They also document a robust and dose-dependent association between RBC transfusion and mortality. However, they failed to demonstrate a significant interaction between the 2 variables.

### Limitations

Our study does present some limitations. First, its observational nature qualifies for demonstrating association rather than causality. Despite controlling for confounding variables with a sophisticated IPW algorithm, we cannot account for those that were not measured. Second, treating RBC transfusion as a dichotomic variable precludes establishing a dose-dependent response that further supports our causality contention. Third, exploring an etiologic classification of preoperative anaemia might help refine the management of these patients [5].

## CONCLUSION

This longitudinal, population-level study extensively emphasizes the adverse short- and long-term outcomes of preoperative anaemia and perioperative RBC transfusion. It stresses the importance of an evidence-based, multimodal and multidisciplinary approach to conserving blood resources and optimizing outcomes in patients at high risk for transfusion—a Patient Blood Management program. In the face of the information conveyed, identifying and optimizing scheduled patients for cardiac surgery at risk and non-arbitrary but evidence-based use of a precious resource should be considered standard practice.

## Supplementary Material

ezad403_Supplementary_Data

## Data Availability

The paper’s data will be shared on reasonable request to the corresponding author.

## ABBREVIATIONS

CABG: Coronary artery bypass grafting
CI: Confidence interval
IPW: Inverse probability-weighted
IQR: Interquartile range
OR: Odds ratio
RBC: Red blood cell
SMD: Standardized mean difference
